# How Does Endothelial Permeability Affect the Development of Juvenile Idiopathic Arthritis? Vascular Endothelial Cadherin as a Promising New Tool Helpful in the Diagnostic Process

**DOI:** 10.1155/2020/8899061

**Published:** 2020-10-21

**Authors:** Krzysztof Orczyk, Elzbieta Smolewska

**Affiliations:** Department of Pediatric Cardiology and Rheumatology, Medical University of Lodz, Sporna 36/50, 91-738 Lodz, Poland

## Abstract

**Introduction:**

Vascular endothelial cadherin (VE-cadherin) is a calcium-dependent protein essential for stabilization of the adherens junctions of the endothelial cells. Through vasculogenic mimicry, VE-cadherin may influence angiogenesis in synovial fibroblast-like cells. The soluble extracellular domain of VE-cadherin may be considered an indicator of endothelial dysfunction. Its potential as a diagnostic biomarker in rheumatic diseases, including juvenile idiopathic arthritis (JIA), needs to be investigated.

**Materials and Methods:**

The study group included 80 patients diagnosed with JIA. In 53 individuals, blood samples were obtained twice with an average interval of 102.4 ± 4.6 days. Results from the study group were compared to 29 age- and sex-matched healthy children.

**Results:**

Serum levels of VE-cadherin were significantly higher in JIA patients than in healthy controls. In such comparison, VE-cadherin had 87.5% sensitivity and 69.0% specificity for the cutoff level 4.36 ng/ml (Youden index 0.56, area under the curve 0.724). VE-cadherin concentrations negatively correlated with the disease activity score. However, such finding may be a false result because of the downregulation of VE-cadherin induced by glucocorticosteroids.

**Conclusions:**

VE-cadherin may become a promising diagnostic biomarker of early stages of JIA. Its predictive significance may be decreased by utilization of glucocorticosteroids. A multicentre study including patients with other arthritides is recommended for further evaluation of this protein.

## 1. Introduction

Maintaining endothelial integrity is a crucial element of building a barrier between blood and surrounding tissues. Destabilization of this barrier leads to leakage of plasma constituents from the vessels which may result in the development of inflammation and tissue edema [1]. Normal functioning of the endothelium is highly reliant on calcium-dependent adhesion membrane proteins called cadherins [2]. Endothelial cells generally express two types of these proteins: neural cadherin (N-cadherin), which is scattered around the cell surface and forms junctions between the basal membrane of the endothelium and underlying pericytes [2], and vascular endothelial cadherin (VE-cadherin), which is mostly responsible for the mediation of complex cell-cell interactions [3]. Extracellular domains of VE-cadherin bind with each other forming zipper-like supercomplexes which provide integrity between adherens junctions [4]. Cytoplasmic tails strengthen the cell-cell adhesion by interacting with intracellular proteins, including beta-catenin, plakoglobin, and p120 [5]. Moreover, VE-cadherin triggers expression of genes which significantly affect endothelial stabilization, specifically claudin-5 and vascular endothelial protein tyrosine phosphatase [6]. Studies on the development of pathological vasculature revealed that VE-cadherin levels strongly correspond with the intensity of angiogenesis in ovarian carcinoma [6] and multiple myeloma [7]. Furthermore, Yamaguchi et al. [8] postulated that fibroblast-like synoviocytes share such VE-cadherin dependency through vasculogenic mimicry; therefore, overexpression of VE-cadherin may affect the development of the vasculature in the synovium in rheumatoid arthritis (RA) patients. Additionally, Bouillet et al. [1] reported elevated titers of autoantibodies against VE-cadherin in RA patients.

**Figure 1:**
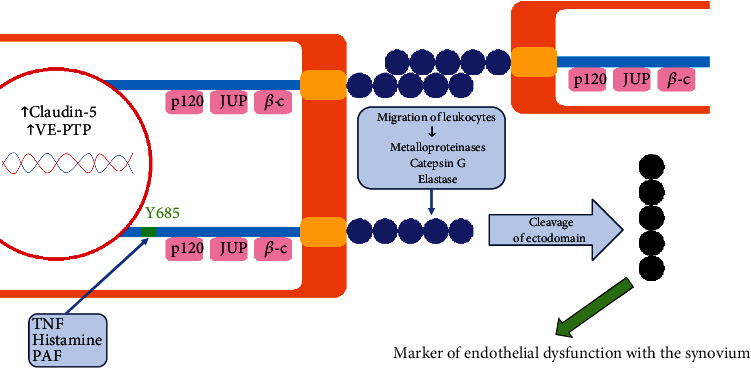
Functioning of VE-cadherin within inflamed tissues. The extracellular domain of VE-cadherin may be released after tyrosine phosphorylation at site Y685 in its cytoplasmic tail, which may be induced by triggers, e.g., TNF, histamine, and PAF. It may also be proteolyzed by enzymes from activated leukocytes, e.g., metalloproteinases, cathepsin G, and elastase. *β*-c: beta-catenin; JUP: junction plakoglobin; PAF: platelet-activating factor; TNF: tumor necrosis factor; VE-cadherin: vascular endothelial cadherin; VE-PTP: vascular endothelial protein tyrosine phosphatase.

VE-cadherin may function in three ways: endocytosis of the full protein and redistribution within the cellular membrane (which are unlikely to be evaluated in clinical conditions) or shedding of the extracellular domain into bloodstream (which has been summarized in Figure 1) [9]. The released extracellular domain of VE-cadherin may be considered a marker of endothelial dysfunction leading to vascular leakage [9]. Cleavage of the ectodomain may be signaled by tyrosine phosphorylation at site Y685 in its cytoplasmic tail [10]. Pathological concentration of tumor necrosis factor (TNF) is a particularly significant inducer of such posttranslational processing of VE-cadherin [11], and other potential triggers include elevated levels of histamine [12] and platelet-activating factor [13]. Furthermore, VE-cadherin may be proteolyzed by metalloproteinases, cathepsin G, and elastase released by activated leukocytes [14, 15]. Therefore, degradation of VE-cadherin plays an important role in the migration of leukocytes into the inflamed tissues [3]. Plasma levels of VE-cadherin were significantly higher in sepsis patients [16] and in the familial Mediterranean fever attack period group [17].

Despite the postulated role of VE-cadherin in the development of RA, serum concentrations of this protein were not yet evaluated in children with juvenile idiopathic arthritis (JIA), which is the most common arthropathy in childhood. The diagnostic process and monitoring of JIA patients still require reliable biomarkers which may significantly influence the aggressiveness of the therapy or the duration of symptoms before making a final diagnosis. The principal objective of the study was to assess whether VE-cadherin may answer any of such necessities.

## 2. Materials and Methods

The study group consisted of 80 patients with JIA: 54 females and 26 males. They were diagnosed at mean age of 7.75 ± 4.27 years and included in the study at mean age of 10.40 ± 4.38 years. Fifty-three children were reassessed after 102.4 ± 26.0 days in order to evaluate the dynamics of the disease activity and the measured parameters. The developed database included the following: active joint count; results of the laboratory tests of inflammatory markers—C-reactive protein (CRP) and erythrocyte sedimentation rate (ESR); and details of current treatment, specifically usage of disease-modifying antirheumatic drugs, intra-articular and systemic glucocorticosteroids (GCS), and biological agents (etanercept, adalimumab, or tocilizumab).

The patients were divided into subgroups regarding the following: (1) JIA subtype according to the International League of Associations for Rheumatology (ILAR) classification [18] (patients with systemic JIA were not included in the study due to its distinct pathogenesis), (2) reason of admission (new diagnosis of JIA, disease flare, continuation of biological treatment, or check-up visit), and (3) disease activity level assessed using the Juvenile Arthritis Disease Activity Score 27-Joint Reduced Count (JADAS27) [19, 20]. In order to make the results more comparable, patients with enthesitis-related arthritis were evaluated using cutoff levels designed for oligo- and polyarticular subtypes, depending on the active joint count. Quantities of each subgroup are summarized in Table 1.

Collected blood samples were stored at -80°C in order to measure serum concentrations of the soluble fraction of VE-cadherin using the ELISA Kit SEB366Hu (CloudClone, China). The obtained results were compared to the control group, which involved 29 age- and sex-matched children who were admitted to the department because of functional cardiovascular system dysfunction.

The statistical analysis was performed using Statistica 13.1 software (StatSoft Polska, Krakow, Poland). The values were presented as mean ± standard deviation (SD). The normality of continuous variables was assessed by the Shapiro-Wilk test. Spearman's rank correlation test was carried out for variables which were not normally distributed. Group comparisons were performed using the Mann-Whitney *U* test and Kruskal-Wallis test. Multivariate analyses involved Wilks' lambda test and subsequent post hoc evaluation with the Tukey honest significant difference test. The receiver operating characteristic (ROC) curve was computed for VE-cadherin, and the area under the curve (AUC) was calculated to evaluate its diagnostic and prognostic usefulness. *p* values lower than 0.05 were considered significant.

The study was approved by the Bioethics Committee of the Medical University of Lodz (approval no. RNN/31/17/KE). All methods were performed in accordance with relevant guidelines and regulations. The informed consent was obtained from parents of all patients.

## 3. Results

Serum concentrations of VE-cadherin were higher in JIA patients than in the control group (6.69 ± 2.13 ng/ml vs. 5.13 ± 2.86 ng/ml, *p* < 0.001). VE-cadherin levels remained elevated at the second measurement (5.57 ± 2.10 ng/ml), but the results did not reach statistical significance (*p* = 0.11). Levels of the measured biomarker did not significantly differ between JIA subtypes (*p* = 0.057 at the study baseline and *p* = 0.51 at the second measurement, respectively).

**Figure 2:**
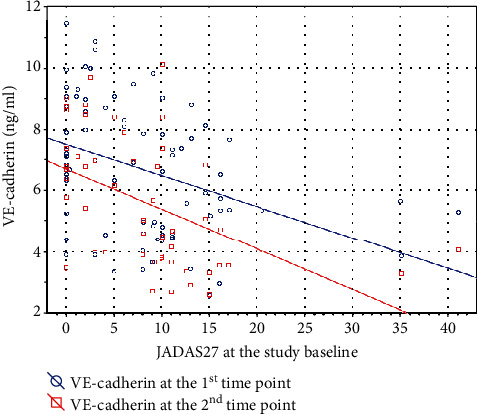
Correlation between the disease activity score and serum levels of VE-cadherin. VE-cadherin: vascular endothelial cadherin; JADAS27: Juvenile Arthritis Disease Activity Score 27-Joint Reduced Count.

**Figure 3:**
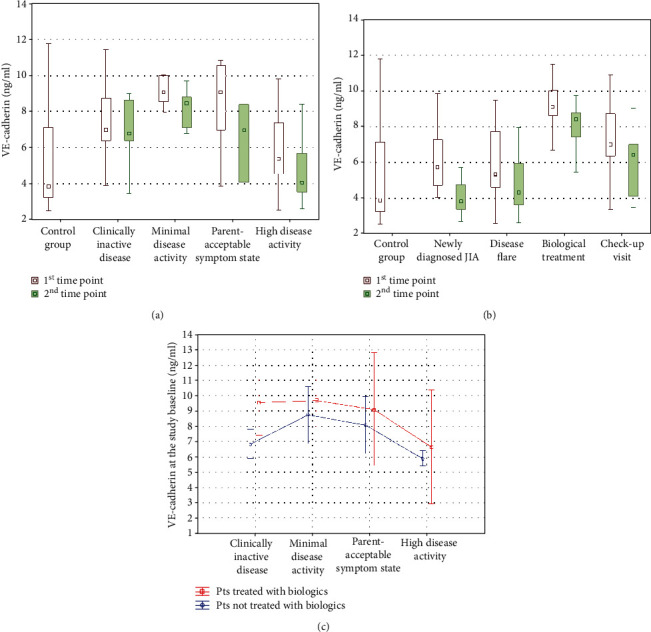
Serum concentrations of VE-cadherin depending on (a) disease activity, (b) reason of admission, and (c) both disease activity and usage of biologics. Pts: patients; VE-cadherin: vascular endothelial cadherin.

Concentrations of VE-cadherin negatively correlated with JADAS27 (*r* = −0.375 and *p* < 0.001 at the study baseline and *r* = −0.335 and *p* = 0.0141 at the second measurement, Figure 2). Dependence of VE-cadherin values on the disease activity remained remarkable in the group assessment (*p* ≤ 0.001 at both time points, Figure 3(a)). Moreover, it also affected the comparison regarding the reason of admission. Patients newly diagnosed with JIA and admitted due to exacerbation of the disease, presumably with higher disease activity than children in the remaining subgroups, had lower VE-cadherin levels (*p* < 0.001 at both measurements, Figure 3(b)). However, multivariate analyses did not reveal any significant influence of inflammatory markers (namely, CRP and ESR) on VE-cadherin levels at any time point.

**Figure 4:**
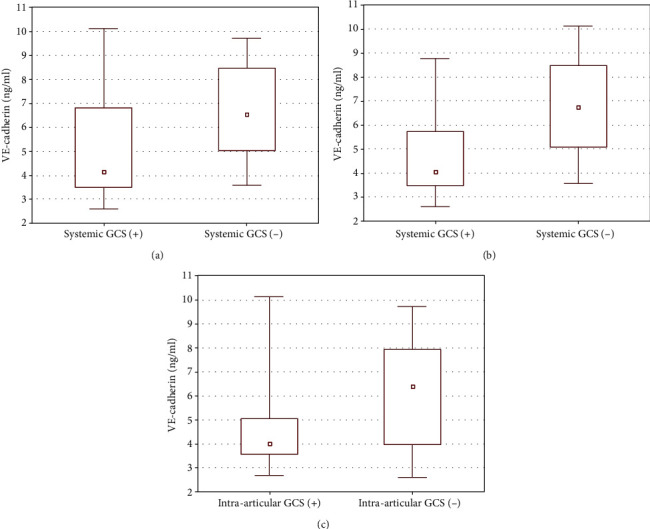
Serum concentrations of VE-cadherin: (a) at the study baseline depending on the usage of systemic GCS, (b) at the second measurement depending on the usage of systemic GCS at the study baseline, and (c) at the second measurement depending on the usage of intra-articular GCS at the study baseline. VE-cadherin: vascular endothelial cadherin; GCS: glucocorticosteroids.

All patients included in the study group were treated with methotrexate; therefore, this parameter was not analyzed. Interestingly, there was no remarkable difference in VE-cadherin concentrations between patients treated and not treated with biologics (Figure 3(c)). Nevertheless, usage of GCS did remarkably influence the obtained results. Forty-three JIA patients (53.8%) required systemic GCS at the study baseline, whereas intra-articular GCS were utilized during the first visit in twenty-four children (30.0%). At the first measurement, VE-cadherin levels were significantly decreased in patients using GCS (*p* = 0.00296, Figure 4(a)). Lower VE-cadherin concentrations at the second time point were observed in patients with positive history of both systemic (*p* = 0.00485, Figure 4(b)) and intra-articular (*p* = 0.131, Figure 4(c)) administration during the first hospitalization.

**Figure 5:**
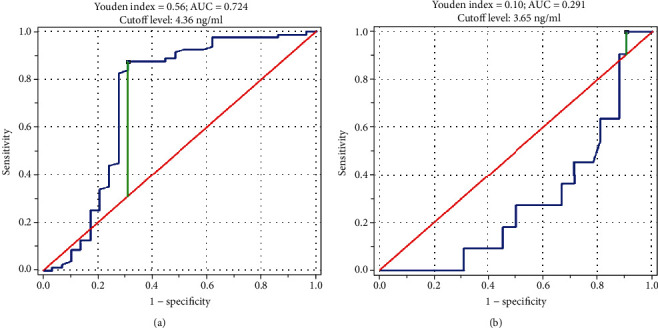
Receiver operating characteristic curve for VE-cadherin as (a) a diagnostic biomarker of JIA and (b) a prognostic marker of developing a disease flare within a 3-month observation. VE-cadherin: vascular endothelial cadherin; JIA: juvenile idiopathic arthritis; AUC: area under the curve.

For the comparison between JIA patients and healthy controls, VE-cadherin was characterized with 87.5% sensitivity and 69.0% specificity for the cutoff level 4.36 ng/ml (Youden index 0.56, AUC 0.724, Figure 5(a)). When considered a prognostic marker of developing a disease flare within a 3-month observation, VE-cadherin had very low specificity 0.095% with 100% sensitivity for the cutoff level 3.65 ng/ml (Youden index 0.10, AUC 0.291, Figure 5(b)). Concentration of VE-cadherin was higher at the second measurement in 13 patients (24.5%), whereas simultaneous elevation of JADAS27 was observed only in 4 of them.

## 4. Discussion

To our best knowledge, this is the first study evaluating the significance of measuring serum concentrations of VE-cadherin in JIA patients. Previous reports involving adults with RA suggested potential usefulness of this biomarker. Sidibe et al. [21] reported that concentration of VE-cadherin was correlated with the disease activity score at the very early stages of RA. Conversely, VE-cadherin levels and anti-VE-cadherin titers were not associated with the disease activity score in the study conducted by Banse et al. [22]. The recent study showed a negative correlation between the VE-cadherin level and disease activity score (JADAS27). Nevertheless, such tendency may be a false significant finding generated by the relevant influence of GCS. As they can decrease endothelial permeability, Banse et al. [22] postulated that GCS therapy may downregulate concentrations of VE-cadherin. In the presented study, patients who were treated with the highest doses of GCS (precisely, children newly diagnosed with JIA or patients admitted to the hospital because of the disease flare) had remarkably lower VE-cadherin levels than patients treated with biological agents, who mostly presented with the low disease activity and did not require GCS. The observed impact of GCS therapy may be considered a potential explanation of discrepancy between results of the study and findings of Sidibe et al. [21] who tested treatment-naїve patients only. Additionally, researchers assessing the relevance of VE-cadherin in RA patients [21, 22] raised the topic of higher risk of cardiovascular events in that population which may be an important confounding factor in analyses regarding VE-cadherin in adult patients.

Elsewhere, the authors reported promising results for another biomarker S100A12 (AUC 0.787 as a diagnostic biomarker and AUC 0.372 as an indicator of a disease flare within a 3-month observation), and its values were not dependent on GCS therapy [23]. Nonetheless, given that the most recent guidelines of long-term care [24] recommend against adding chronic low-dose GCS in JIA patients regardless of the disease activity, such drawback of VE-cadherin may lose its importance in the future research.

Banse et al. [22] showed a significant correlation between VE-cadherin and CRP at different time points. On the contrary, Sidibe et al. [21] did not observe any relationship between VE-cadherin and inflammatory markers. In the recent study, neither CRP nor ESR was linked to VE-cadherin level. Moreover, Sidibe et al. [21] suggested that VE-cadherin was not associated with the “classical” serological markers (namely, rheumatoid factor and anticitrullinated protein antibodies). The authors were unable to verify such finding as the presence of rheumatoid factor was detected in only one patient.

Additionally, literature data suggested potential influence of vitamin D on vascular permeability. In the study published by Vila Cuenca et al. [25], paricalcitol (which is an active vitamin D analogue) directly mediated endothelial integrity *in vitro* by enforcing cell-cell adhesion. However, in the recent study, vitamin D insufficiency did not influence VE-cadherin levels (data not shown).

Interestingly, VE-cadherin is not the only endothelial protein affecting the development of inflammation within the course of JIA. Vascular endothelial growth factor (VEGF) is the best recognized and the most endothelial-specific angiogenic factor, and its higher concentrations in serum and synovial fluid were reported in JIA patients [26]. However, the authors were unable to measure VE-cadherin levels in synovial fluid in the recent study. As antiangiogenic therapy has become a promising novel perspective for the treatment of RA, clinical trials should consider not only anti-VEGF antibodies (such as bevacizumab and vatalanib) [27] but also drugs affecting VE-cadherin levels like tyrosine-kinase inhibitors (such as sunitinib) [10].

The main limitation of the study was a lack of comparison with patients with other inflammatory arthritides, which could supplement the evaluation of real usefulness of VE-cadherin in differential diagnosis of JIA. Furthermore, the relative heterogeneity of the included patients was another drawback of the study. JIA is a relatively rare disease; therefore, it is difficult to form a consistent study group in one academic centre. Furthermore, the overtreatment with systemic GCS should be eliminated in order to limit the potential side effects and evaluate the recommended treatment more effectively.

## 5. Conclusions

VE-cadherin presents a promising potential of becoming a diagnostic biomarker of JIA. Its predictive value as a marker of disease flare depends on the usage of GCS. The possible usefulness of the biomarker has to be validated in further research including patients with other arthritides.

## Figures and Tables

**Table 1:** General characteristics of the study group. JIA: juvenile idiopathic arthritis; RF: rheumatoid factor; ERA: enthesitis-related arthritis; JADAS27: Juvenile Arthritis Disease Activity Score 27-Joint Reduced Count.

	1^st^ time point (*n* = 80)	2^nd^ time point (*n* = 53)
JIA subtypes, *n* (%)		
Oligoarticular JIA	54 (67.5%)	35 (66.0%)
RF-negative poly-JIA	11 (13.75%)	9 (17.0%)
RF-positive poly-JIA	1 (1.25%)	1 (1.9%)
ERA	14 (17.5%)	8 (15.1%)
Reason of admission, *n* (%)		
New diagnosis of JIA	16 (20.0%)	—
Disease flare	26 (32.5%)	11 (20.8%)
Continuation of biological treatment	9 (11.25%)	12 (22.6%)
Check-up visit	29 (36.25%)	30 (56.6%)
Disease activity level (according to JADAS27), *n* (%)		
Clinically inactive disease	19 (23.75%)	16 (30.2%)
Minimal disease activity	7 (8.75%)	12 (22.65%)
Parent-acceptable symptom state	5 (6.25%)	6 (11.3%)
High disease activity	49 (61.25%)	19 (35.85%)

## Data Availability

The data used to support the findings of this study are included within the article. The supplementary data are available from the corresponding author upon request.

## References

[1] Bouillet L., Baudet A. E., Deroux A. (2013). Auto-antibodies to vascular endothelial cadherin in humans: association with autoimmune diseases. *Laboratory Investigation*.

[2] Colas-Algora N., Millan J. (2019). How many cadherins do human endothelial cells express?. *Cellular and Molecular Life Sciences*.

[3] Brasch J., Harrison O. J., Ahlsen G. (2011). Structure and binding mechanism of vascular endothelial cadherin: a divergent classical cadherin. *Journal of Molecular Biology*.

[4] Dejana E., Orsenigo F., Lampugnani M. G. (2008). The role of adherens junctions and VE-cadherin in the control of vascular permeability. *Journal of Cell Science*.

[5] Sidibé A., Polena H., Pernet-Gallay K. (2014). VE-cadherin Y685F knock-in mouse is sensitive to vascular permeability in recurrent angiogenic organs. *American Journal of Physiology. Heart and Circulatory Physiology*.

[6] Morini M. F., Giampietro C., Corada M. (2018). VE-cadherin-mediated epigenetic regulation of endothelial gene expression. *Circulation Research*.

[7] Wrobel T., Mazur G., Wolowiec D., Jazwiec B., Sowinska E., Kuliczkowski K. (2006). sVE-cadherin and sCD146 serum levels in patients with multiple myeloma. *Clinical and Laboratory Haematology*.

[8] Yamaguchi K., Sudo H., Imai K. (2019). Vascular endothelial growth factor signaling in VE-cadherin expression and tube-like formation by rheumatoid arthritic synovial fibroblast-like cells. *Biochemical and Biophysical Research Communications*.

[9] Zhang R., Li R., Tang Y. (2019). Soluble vascular endothelial cadherin: a promising marker of critical illness?. *Critical Care*.

[10] Polena H., Creuzet J., Dufies M. (2018). The tyrosine-kinase inhibitor sunitinib targets vascular endothelial (VE)-cadherin: a marker of response to antitumoural treatment in metastatic renal cell carcinoma. *British Journal of Cancer*.

[11] Angelini D. J., Hyun S. W., Grigoryev D. N. (2006). TNF-alpha increases tyrosine phosphorylation of vascular endothelial cadherin and opens the paracellular pathway through fyn activation in human lung endothelia. *American Journal of Physiology. Lung Cellular and Molecular Physiology*.

[12] Shasby D. M., Ries D. R., Shasby S. S., Winter M. C. (2002). Histamine stimulates phosphorylation of adherens junction proteins and alters their link to vimentin. *American Journal of Physiology. Lung Cellular and Molecular Physiology*.

[13] Hudry-Clergeon H., Stengel D., Ninio E., Vilgrain I. (2005). Platelet-activating factor increases VE-cadherin tyrosine phosphorylation in mouse endothelial cells and its association with the PtdIns3'-kinase. *The FASEB Journal*.

[14] Carden D., Xiao F., Moak C., Willis B. H., Robinson-Jackson S., Alexander S. (1998). Neutrophil elastase promotes lung microvascular injury and proteolysis of endothelial cadherins. *The American Journal of Physiology*.

[15] Herren B., Levkau B., Raines E. W., Ross R. (1998). Cleavage of beta-catenin and plakoglobin and shedding of VE-cadherin during endothelial apoptosis: evidence for a role for caspases and metalloproteinases. *Molecular Biology of the Cell*.

[16] Yu W.-K., McNeil J. B., Wickersham N. E., Shaver C. M., Bastarache J. A., Ware L. B. (2019). Vascular endothelial cadherin shedding is more severe in sepsis patients with severe acute kidney injury. *Critical Care*.

[17] Yel S., Dursun İ., Çetin F. (2018). Increased circulating endothelial microparticles in children with FMF. *Biomarkers*.

[18] Petty R. E., Southwood T. R., Manners P. (2004). International League of Associations for Rheumatology classification of juvenile idiopathic arthritis: second revision, Edmonton, 2001. *The Journal of Rheumatology*.

[19] Consolaro A., Bracciolini G., Ruperto N. (2012). Remission, minimal disease activity, and acceptable symptom state in juvenile idiopathic arthritis: defining criteria based on the juvenile arthritis disease activity score. *Arthritis and Rheumatism*.

[20] Consolaro A., Ruperto N., Bracciolini G. (2014). Defining criteria for high disease activity in juvenile idiopathic arthritis based on the juvenile arthritis disease activity score. *Annals of the Rheumatic Diseases*.

[21] Sidibé A., Mannic T., Arboleas M. (2012). Soluble VE-cadherin in rheumatoid arthritis patients correlates with disease activity: evidence for tumor necrosis factor *α*-induced VE-cadherin cleavage. *Arthritis and Rheumatism*.

[22] Banse C., Polena H., Stidder B. (2017). Soluble vascular endothelial (VE) cadherin and autoantibodies to VE-cadherin in rheumatoid arthritis patients treated with etanercept or adalimumab. *Joint, Bone, Spine*.

[23] Orczyk K., Smolewska E. (2018). A granulocyte-specific protein S100A12 as a potential prognostic factor affecting aggressiveness of therapy in patients with juvenile idiopathic arthritis. *Journal of Immunology Research*.

[24] Ringold S., Angeles-Han S. T., Beukelman T. (2019). 2019 American College of Rheumatology/Arthritis Foundation guideline for the treatment of juvenile idiopathic arthritis: therapeutic approaches for non-systemic polyarthritis, sacroiliitis, and enthesitis. *Arthritis Care Res (Hoboken)*.

[25] Vila Cuenca M., Ferrantelli E., Meinster E. (2018). Vitamin D attenuates endothelial dysfunction in uremic rats and maintains human endothelial stability. *Journal of the American Heart Association*.

[26] Świdrowska J., Smolewski P., Stańczyk J., Smolewska E. (2015). Serum angiogenesis markers and their correlation with ultrasound-detected synovitis in juvenile idiopathic arthritis. *Journal of Immunology Research*.

[27] Świdrowska-Jaros J., Smolewska E. (2018). A fresh look at angiogenesis in juvenile idiopathic arthritis. *Cent Eur J Immunol.*.

